# The association between circulating 25-hydroxyvitamin D and cardiovascular diseases: a meta-analysis of prospective cohort studies

**DOI:** 10.1186/s12872-019-1236-7

**Published:** 2019-11-07

**Authors:** Fatemeh Gholami, Ghobad Moradi, Bushra Zareei, Mohammad Aziz Rasouli, Bahram Nikkhoo, Daem Roshani, Ebrahim Ghaderi

**Affiliations:** 1 Department of Epidemiology, School of Public Health, Iran University of Medical Sciences, Tehran, Iran; 2 Social Determinants of Health Research Center, Research Institute for Health Development, Kurdistan University of Medical Sciences, Sanandaj, Iran; 3 Department of Epidemiology and Biostatistics, Faculty of Medicine, Kurdistan University of Medical Sciences, Sanandaj, Iran; 4 Vice Chancellor for Educational and Research, Clinical Research Development Unit, Kowsar Hospital, Kurdistan University of Medical Sciences, Sanandaj, Iran; 5 Department of Pathology, Faculty of Medicine, Kurdistan University of Medical Sciences, Sanandaj, Iran

**Keywords:** Vitamin D, Cardiovascular disease, Meta-analysis, Prospective cohort study

## Abstract

**Background:**

There is a controversy about the association between vitamin D and cardiovascular diseases (CVDs). The effect of serum 25-OH-vitD on the risk of CVDs was evaluated.

**Methods:**

Major electronic databases including Scopus, Science Direct, and PubMed were searched. All prospective cohort studies on the relationship between vitamin D status and CVDs conducted between April 2000 and September 2017 were included, regardless language. The study participants were evaluated regardless of their age, sex, and ethnicity. The Newcastle-Ottawa Scale was used to assess the quality of the studies. Two investigators independently selected the studies and extracted the data. The designated effects were risk ratio (RR) and hazard ratio (HR). The random effects model was used to combine the results.

**Results:**

A meta-analysis of 25 studies with 10,099 cases of CVDs was performed. In general, a decrease in the level of vitamin D was associated with a higher relative risk of CVDs (incidence-mortality combined) (RR = 1.44, 95% CI: 1.24–1.69). This accounts for 54% of CVDs mortality rate (RR = 1.54, 95% CI: 1.29–1.84(. However, no significant relationship was observed between the vitamin D status and incidence of CVDs (RR = 1.18, 95% CI: 1–1.39). In general, low serum vitamin D level increased the risk of CVD by 44% (RR = 1.44, 95% CI: 1.24–1.69). It also increased the risk of CVD mortality (RR = 1.54, 95% CI: 1.29–1.84) and incidence rates (RR = 1.18, 95% CI: 1–1.39).

**Conclusions:**

The findings showed that vitamin D deficiency increases the CVDs mortality rate. Due to the limited number of studies on patients of the both genders, further research is suggested to separately evaluate the effect of vitamin D status on CVD in men and women.

## Background

Vitamin D may play a role in the pathogenesis of several extra-skeletal disorders involving the dermatological, cardiovascular, immune or metabolic systems [1]. In addition, low vitamin D status is associated with other conditions including osteoporotic fractures, cancer, diabetes, respiratory diseases, and an increased all-cause mortality [2–10]. One of the most important issues is the effect of vitamin D deficiency on the incidence and mortality rates of cardiovascular diseases (CVDs) [11].

The CVDs are one of the top leading causes of mortality and morbidity worldwide [12]. Coronary heart disease (CHD) accounts for more than one half of the deaths in the developing countries and one-fourth of the deaths in the developed countries [12, 13]. The CHD is the most common type of heart disease, and the annual direct and indirect medical costs associated with it exceed $100 billion each year in the United States [14].

A reduced level of 25-hydroxyvitamin D (25-OH-vitD) in plasma, as an indicator of vitamin D deficiency, is associated with several risk factors of stroke, such as hypertension, thrombosis, atherosclerosis, and inflammation. A few studies have shown that the reduction in 25-OH-vitD is directly associated with an increased risk of stroke and CVDs. However, some studies have shown completely different results [15–21]. Previous meta-analysis results from Randomized Controlled Trial (RCTs) have shown that the effects of vitamin D supplementation on CVDs, the risk factors, or glycemic outcomes are controversial or null [22]. Due to a lack of consensus on the association between serum 25-OH-vitD and CVDs, there is no established and clear measure for protecting against vitamin D deficiency and reducing the incidence and mortality of CVDs. Therefore, this study is aimed to determine the relationship between the level of 25-OH-vitD and CVDs incidence and mortality in people without underlying CVD-related conditions.

## Methods

The authors are asked to provide registration information about the systematic review, (registration number: 116885).

**Fig. 1:**
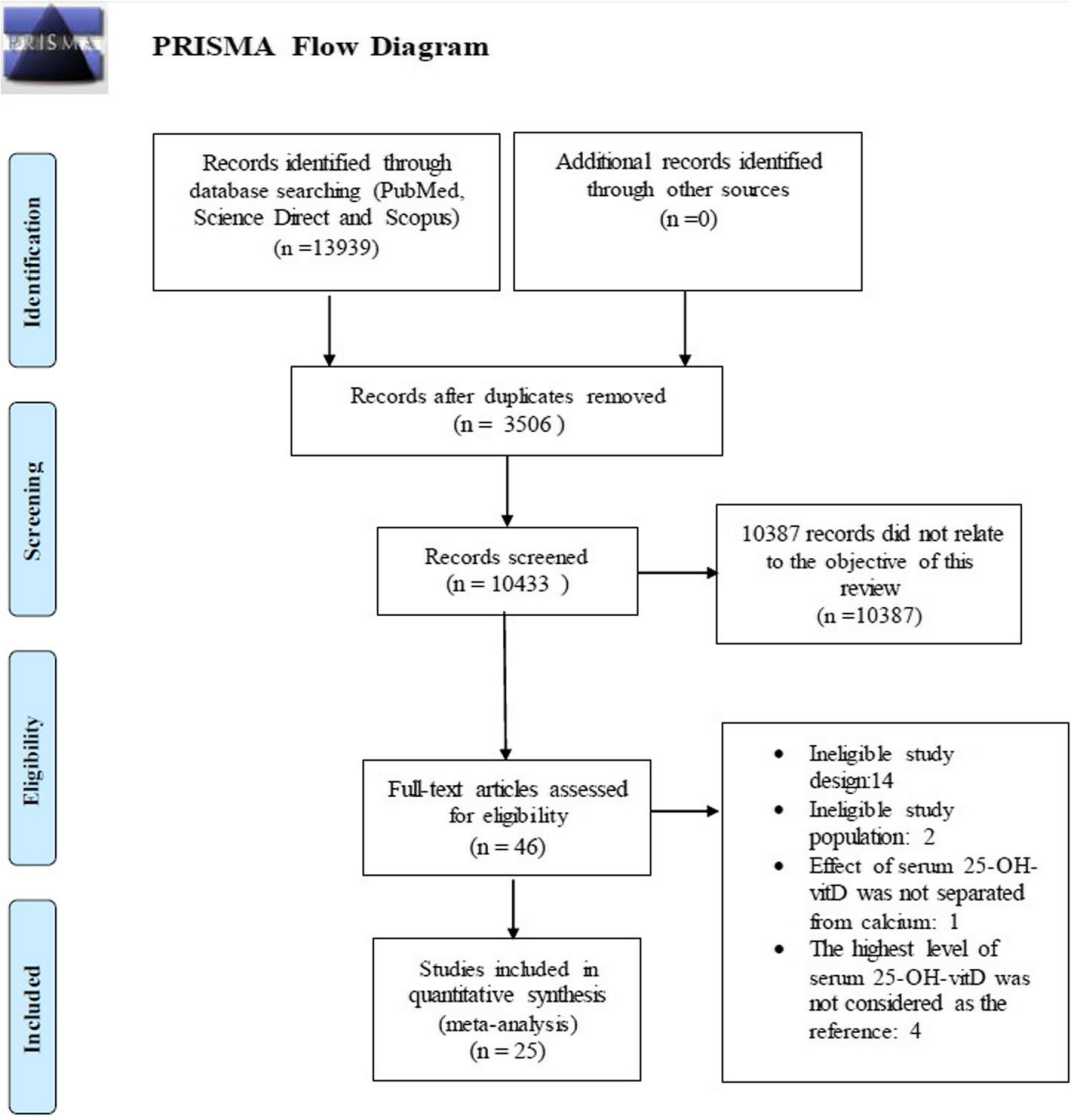
Flow diagram of the study selection process. As shown our initial searches resulted in 13,939 citations. After screening title and abstracts, 46 studies were considered potentially eligible and retrieved in full text, of these 25 studies were subsequently included in the meta-analysis

This systematic review and Meta-analysis was performed according to the Meta-Analyses of Observational Studies in Epidemiology (MOOSE) and Preferred Reporting Items for Systematic Reviews and Meta-Analyses (PRISMA) (RRR) [23, 24]. (Fig. 1).

### Information sources

Three databases were searched including PubMed (April 2000 to September 2017), Science Direct (April 2000 to September 2017), and Scopus (April 2000 to September 2017). In order to find additional references, the reference lists of all retrieved studies, especially systematic reviews were also scanned [25–31]. Furthermore theses and journals about the association of vitamin D and CVDs were also searched.

### Search

Prospective cohort studies on people without underlying CVD-related conditions addressing the association between vitamin D and CVDs were included. The search strategy included a combination of the following keywords:

“vitamin D” OR “cholecalciferol” OR “25-hydroxyvitamin D” OR “25-OH-D” OR “25(OH)D” AND “cerebrovascular disease” OR “cardiovascular diseases” AND “mortality” OR “incidence” OR “survival” AND “prospective studies” OR “cohort studies” OR “longitudinal studies” OR “observational studies.”

### Study selection

All prospective cohort studies that evaluated the relationship between vitamin D and CVDs were included in the study, regardless of their language. The study population consisted of healthy individuals regardless of age, gender, and ethnicity. Only studies published after 2000 were included because the methods of measuring Vitamin D in these studies were different from those published before 2000. The exclusion criteria were 1) studies whose baseline population had diseases like metabolic syndrome, diabetes, and CVD, or needed hemodialysis; 2) case reports, editorials, letters, meeting abstracts, or review articles; and 3) retrospective studies, cross-sectional studies, or case-control studies. Exposure was defined as the level of vitamin D, and the outcome of the study was the incidence of CVDs or mortality due to CVDs. The CVDs included in the study were stroke and CHD [WHO International Classification of Diseases (ICD)-10 I60–69; http://www.who.int/classifications/icd/en]. In addition, cardiac arrest (I46), heart failure (I50), and sudden death (R69) were included. The CHD included acute myocardial infarction, angina pectoris, and other ischemic heart diseases (ICD- 10 I20-I25).

To ensure correct paper selection according to the inclusion criteria, two researchers (FG and MAR) conducted the selection process independently. They were not blind to the name of the authors, the journals, and the results. Any disagreement between them would be resolved by consulting with the third researcher (GM).

Full-text of the selected titles were retrieved and assessed by two authors (FG, MAR) independently to ensure adherence to selection criteria. In addition, the reference lists of the studies were searched to identify additional publications.

### Screening, data extraction, and quality assessment

The primary search results were reviewed and duplicate studies were deleted. In addition, more articles were eliminated after reviewing the titles and abstracts. Finally, full text of the remaining articles was assessed for eligibility. Two researchers (FG, MAR) extracted the data from the selected studies. The extracted variables for data analysis included the name of the first author, study title, publication year, study location, participants’ age at baseline, sample size, number of cases, follow-up duration, gender, study outcomes (incidence of or mortality due to CVD), levels of circulating 25-OH-vitD (values in ng/ml multiplied by 2.496 for conversion to nmol/l), measurement methods (radioimmunoassay, mass spectrometry, etc.), and RR (95% CI) for the highest vs. lowest categories of vitamin D and variables adjusted in the analysis. The extracted data were entered into an electronic data sheet.

The Newcastle-Ottawa Scale (NOS) was used by two independent researchers (GF and MAR) to evaluate the risk of bias and the quality of the studies [32]. This scale is based on a star system (maximum of nine stars) to evaluate a study in three domains: selection of participants, comparability of study groups, and the ascertainment of outcomes of interest. Studies that received a score of nine stars were categorized as low risk of bias (high quality), seven or eight stars as medium risk (moderate quality), and six or less as high risk of bias (low quality).

### Statistical analysis

Pooled measures were calculated as the inverse variance-weighted mean of the logarithm of RR and HR with 95% CI to assess the strength of the association. The RR (95% CI) for the highest vs. lowest categories of vitamin D was used in this study.

The results were reported separately for men and women and the effect was evaluated using the random effects model. The consistency of findings across studies was assessed by standard χ^2^ tests and the I^2^ statistic [33]. Heterogeneity was evaluated quantitatively using I^2^ according to the Higgins classification in which I^2^ = 25, 50, and 75% indicate low, moderate, and high heterogeneity respectively [34]. A funnel plot [35] and the Egger’s test were used to evaluate publication bias [36].

Sensitivity analysis was performed with one study removed at a time [37], so that a study would be considered excessively influential if the significance of its “omitted” meta-analytic estimate differed relatively from the overall estimate. Meta-regression was used to evaluate the heterogeneity between studies [38]. The data analyses were done in Stata 12 (Stata Corp, College Station, TX) (*P* < 0.05). The authors are asked to provide registration information about the systematic review, (registration number: 116885).

## Results

A total of 13,939 studies were found up to September 2017 of which 845 references were selected through checking the reference lists. Of the 10,433 retrieved references, 3506 references were excluded as duplicates, and 10,387 references were irrelevant. Out of 46 retrieved references with full text, 25 studies were eligible for the meta-analysis.

### Characteristics of the reviewed studies

This meta-analysis was performed on the 25 studies with 10,099 cases of CVDs (Table 1). The incidence rate of CVDs was evaluated in six studies [30, 41, 42, 44, 47, 48], and the mortality rate of CVDs was evaluated in 19 studies. The serum level of 25-OH-vitD was measured using radioimmunoassay, mass spectrometry, and other methods. Radioimmunoassay was used in 10 studies [18, 19, 39, 40, 45, 50, 56, 58–60] and mass spectrometry was used in six studies [44, 46, 47, 49, 52, 54]. The highest level of serum 25-OH-vitD was considered as the reference. In the majority of papers, the highest and lowest categories of vitamin D consumption were 50 nmol/l and 30 nmol/l respectively. Totally, 13 studies were conducted in the US [21, 44–51, 56, 59, 60], 11 in Europe [10, 19, 39–43, 53–55, 58, 61], and one was carried out in another continent [18]. Five studies presented the results for male patients [39, 44, 47, 52, 54], two studies evaluated female patients [18, 50], and other studies included both male and female patients. Moreover, the quality of 18 studies was high and quality of seven studies was intermediate [19, 40, 44, 45, 51, 58, 59]. The follow-up period was 5 years in four studies [40, 41, 47, 51] and longer in other studies (Table 2). The data of all the reviewed studies were adjusted for age and gender.

**Table 1:** Characteristics of studies included on dairy foods and CVD

Author	Study, continent	Age (years)	Subjects	Sex	Quality	Follow-up (years)	Outcome (cases) CVD	Method of measurement (vitamin D)
Lee, 2014 [39]	European Male Ageing Study (EMAS), Europe	60	2452	Male	High	4.3	72	Radioimmunoassay (RIA)
Formiga, 2014 [40]	The Octabaix Study, Europe	85	312	Both	Moderate	2.8	25	Radioimmunoassay (RIA)
Perna, 2013 [41]	ESTHER Study, Europe	50–74	7709	Both	High	6.5	1011	DiaSorin-Liason (Diasorin, Inc) and the IDS-iSYS (Immunodiagnostic Systems GmbH) immunoassayes
Kühn, 2013 [42]	The European Investigation into Cancer and Nutrition (EPIC), Europe	50.6	2132	Both	High	7.7	1030	MassChrom reagent kit
Scho¨ttker, 2013 [43]	ESTHER study, Europe	50–74	9578	Both	High	9.5	350	Diasorin-Liaison analyzer
Bajaj, 2013 [44]	Osteoporotic Fractures in Men (MrOS), American	> 65	2990	Male	Moderate	5.9	740	Liquid chromatography-tandem mass spectrometry
Liu, 2012 [45]	Third National Health and Nutrition Examination Survey (NHANESIII), American	≥ 35	13,134	Both	Moderate	8	1451	Radioimmunoassay (RIA)
Kritchevsky, 2012 [46]	the Health, Aging, and Body Composition study (Health ABC) study, American	74.7	2638	Both	High	8.5	228	Radioimmunoassay (RIA)
Messenger, 2012 [47]	Osteoporotic Fractures in Men (MrOS) Study, American	76.4	813	Male	High	4.4	140	Mass spectrometry (MS)
Welsh, 2012 [48]	The MIDSPAN Family Study, American	30–59	1522	Both	High	14.4	297	Mass spectrometry (MS)
Kestenbaum, 2011 [49]	The CHS (Cardiovascular Health Study), American	≥ 65	2312	Both	High	14	389	Mass spectrometry (MS)
Eaton, 2011 [50]	Women’s Health Initiative (WHI), American	50–79	2429	Female	High	10	79	Radioimmunoassay
Anderson, 2010 [51]	Intermountain Heart Collaborative (IHC), American	55	27,686	Both	Moderate	1.3	1193	Chemiluminescent immunoassay
Bolland, 2010 [18]	Healthy postmenopausal women, Others	74	1471	Female	High	5	63	Radioimmunoassay (RIA)
Cawthon, 2010 [52]	Osteoporotic Fractures in Men (MrOS) study, American	≥ 65	1594	Male	High	7.3	110	Mass spectrometry (MS)
Virtanen, 2010 [53]	The KIHD study, Europe	53–73	1136	Both	High	9.1	35	An HPLC using diode array detector
Michae¨lsson, 2010 [54]	The Uppsala Longitudinal Study of Adult Men (ULSAM), Europe	71	1194	Male	High	12.7	196	Mass spectrometry (MS)
Jassal, 2010 [21]	The Rancho Bernardo Study, American	76	1073	Both	High	6.8	111	Competitive binding protein recognition and chemiluminescence detection
Hutchinson, 2010 [55]	the fourth Tromsø study, Europe	58.9	7161	Both	High	11.7	325	Immunometry (ECLIA) using an automated clinical chemistry analyzer
Ginde, 2009 [56]	National Center for Health Statistics conducted the Third National Health and Nutrition Examination Survey (NHANES III), American	≥ 65	3408	Both	High	7.3	767	Radioimmunoassay (RIA)
Pilz, 2009 [57]	The Hoorn Study Europe	50–75	614	Both	High	6.2	20	Means of a competitive binding protein assay
Semba, 2009 [58]	Invecchiare in Chianti, “Aging in the Chianti Area” (InCHIANTI) study, Europe	≥ 65	1006	Both	Moderate	6.5	107	Radioimmunoassay (RIA)
Dobnig, 2008 [19]	The Ludwigshafen Risk and Cardiovascular Health (LURIC) study, Europe	62	737	Both	Moderate	7.7	463	Radioimmunoassay (RIA)
Melamed, 2008 [59]	Third NationalHealth and Nutrition Examination Survey (NHANESIII), American	≥ 20	1331	Both	Moderate	8.7	777	Radioimmunoassay (RIA)
Wang, 2008 [60]	The Framingham Offspring cohort, American	59	1739	Both	High	5.4	120	Radioimmunoassay (RIA)

**Table 2:** Summary risk estimates of the association between vitamin D and risk of CVD

	N^b^	Number of cases	Risk estimate (95% CI)	Heterogeneity test		
			REM	I^2^ (%)	P	P^a^
Vitamin D	25	10,099	1.44 (1.24–1.69)	84.7	0.00	
Outcome						0.11
Incidence [32, 41, 42, 44, 47, 60]	6	3338	1.18 (1–1.39)	57.7	0.03	
Mortality [18, 19, 21, 39, 40, 43, 45, 46, 49–59]	19	6761	1.54 (1.29–1.84)	81.4	0.00	
Location where the study was conducted						
American [21, 44–52, 56, 59, 60]	13	6402	1.31 (1.12–1.54)	73.8	0.00	1
Europe [19, 39–43, 53–55, 57, 58]	11	3634	1.67 (1.27–2.19)	85.6	0.00	0.09
Others [18]	1	63	0.90 (0.50–1.61)	–	–	–
Sex						0.80
Male [39, 44, 47, 52, 54]	5	1258	1 (0.84–1.20)	0.00	0.54	
Female [18, 50]	2	142	1.12 (0.78–1.59)	0.00	0.35	
Quality						0.26
High [18, 21, 39, 41–43, 46–50, 52–57, 60]	18	5343	1.30 (1.16–1.46)	38.8	0.04	
Moderate [19, 40, 44, 45, 51, 58, 59]	7	4756	1.58 (1.15–2.15)	92.7	0.00	
Measurement of vitamin D						
Radioimmunoassay [18, 19, 39, 40, 45, 50, 56, 58–60]	10	3924	1.61 (1.23–2.11)	82.3	0.00	1
Mass spectrometry [44, 46, 47, 49, 52, 54]	7	2100	1.08 (0.96–1.21)	8.3	0.36	0.03
Others [21, 41–43, 51, 53, 55, 57]	8	6024	1.45 (1.21–1.74)	72.1	0.00	0.39
Duration of follow up						0.98
< 5 years [39, 40, 47, 51]	4	1430	1.69 (1.46–1.96)	0.00	0.39	
> 5 years [18, 19, 21, 41–46, 48–50, 52–60]	21	8669	1.45 (1.22–1.73)	86.7	0.00	

*REM* random effect model

^a^ Pvalue for metaregression, and location where the study was conducted (American as the reference) and Measurement of vitamin D (Radioimmunoassay as the reference)

^b^ N: number of results

### Association between circulating 25-OH-vitD levels and CVDs

The effect of vitamin D status on CVDs was calculated using RR and the effect was considered with the greatest degree of control for the potential confounders. Here, RR (95% CI) for the highest vs. lowest categories of vitamin D was used. In general, decreased level of vitamin D was associated with an increased relative risk of CVDs (RR = 1.44, 95% CI: 1.24–1.69), accounting for 54% of CVDs mortality (RR = 1.54, 95% CI: 1.29–1.84(. However, no significant relationship was observed between vitamin D status and incidence of CVDs (RR =1.18, 95% CI: 1–1.39).

**Fig. 2:**
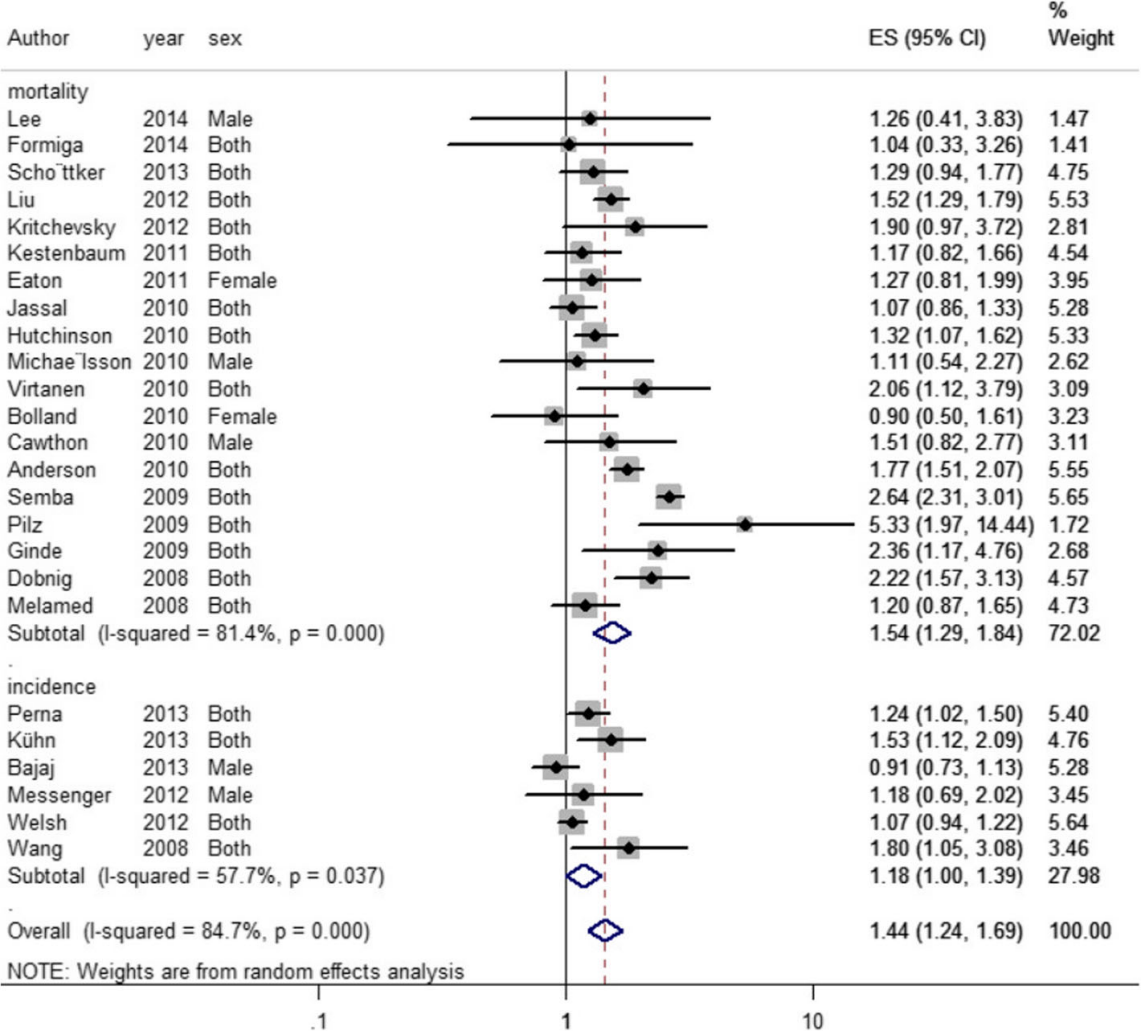
The association of vitamin D with total cardiovascular diseases events, cardiovascular diseases incidence and cardiovascular diseases mortality. The size of gray box is positively proportional to the weight assigned to each study, and horizontal lines represent the 95% confidence intervals

In general, low circulating 25-OH-vitD levels increased the risk of CVDs by 44% (RR = 1.44, 95% CI: 1.24–1.69) (Fig. 1). Low circulating 25-OH-vitD levels increased the risk of CVDs incidence (RR = 1.18, 95% CI: 1–1.39) and mortality (RR = 1.54, 95% CI: 1.29–1.84) (Fig. 1). The size of the gray box was proportional to the weight assigned to each study, and the horizontal lines represent the 95% CIs. There was evidence of heterogeneity (I^2^) between the observational studies, which was equal to 84.7% (*P* < 0.001) for CVD (Fig. 2).

### Subgroup analysis and assessment of publication bias

**Fig. 3:**
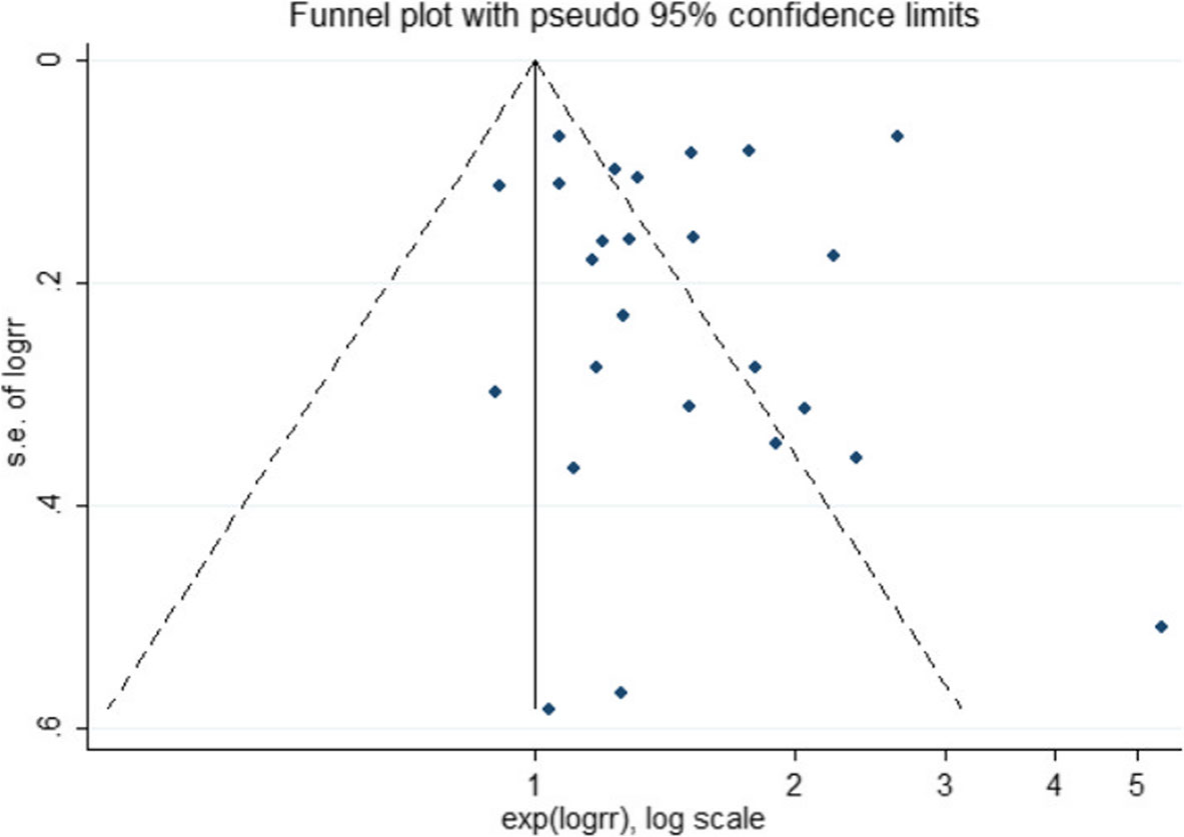
Funnel plot for the analysis of vitamin D and cardiovascular diseases

There was no publication bias according to the Egger’s test (*P* = 0.76). Asymmetry was observed in the funnel plot (Fig. 3), due to small-study effects [62], in a study by Pilz, Lee, and Formiga [39, 40, 61]. There was no change in the overall estimate (RR) of the study after excluding three studies. The results of the sensitivity analysis for CVDs showed that excluding none of the studies changed the overall estimate of the study significantly; this relationship was 1.44, ranging from 1.23 to 1.68.

A meta-regression was performed in the subgroups to detect the source of heterogeneity. The estimated effect showed no association with the outcome [CVD incidence: RR = 1.18 (95% CI: 1–1.39); CVD mortality: RR = 1.54 (95% CI: 1.29–1.84)] (*P* = 0.11); location [Europe: RR = 1.25 (95% CI: 0.95–1.65); others: RR = 0.68 (95% CI: 0.30–1.54)] (*P* = 0.12(; quality [high: RR = 1.30 (95% CI: 1.16–1.46); moderate: RR = 1.58 (95% CI: 1.15–2.15)] (*P* = 0.26); gender [male: RR = 1 (95% CI: 0.84–1.20); female: RR = 1.12 (95% CI: 0.78–1.59)] (*P* = 0.80); and duration of follow-up [< 5 years: RR = 1.69 (95% CI: 1.46–1.96); > 5 years: RR = 1.45 (95% CI: 1.22–1.73)] (*P* = 0.98).

Based on the studies that used mass spectrometry to measure the serum level of 25–OH-vitD (RR = 1.08 (95% CI: 0.96–1.21) and compared with the studies that used radioimmunoassay (RR = 1.61 (95% CI: 1.23–2.11) (*P* = 0.03), there may be an association between serum 25-OH-vitD and an increased risk of CVD by 8% .

The number of subjects (*P* = 0.69) and cases (*P* = 0.91) were not among the sources of heterogeneity (Table 2).

## Discussion

The results of the meta-analysis showed a negative association between the serum 25-OH-vitD concentration and the risk of CVDs morbidity and mortality. Decreased level of vitamin D was associated with an increased relative risk of CVD (RR = 1.44, 95% CI: 1.24–1.69), accounting for 54% of CVD mortality (RR = 1.54, 95% CI: 1.29–1.84(. However, no significant relationship was observed between the vitamin D status and incidence of CVDs (RR = 1.18, 95% CI: 1–1.39).

The results from the experimental studies have shown a positive effect of vitamin D on the risk factors associated with CVDs and its progression [57, 63–66].

The results from the meta-analysis revealed that the majority of the cohort studies found a significant relationship between the vitamin D status and CVD mortality rate [26, 67, 68], which is consistent with the previous reviews. However, some studies found no linear relationship between the vitamin D status and the risk of CVDs [59, 60]. Moreover, the results from the cohort studies revealed that the patients who received more than 75–87.5 nmol/l of 25-OH-vitD were more likely to die due to CVDs [69], which may be due to the limited number of studies included in the meta-analysis as well as the application of different levels of vitamin D and adjustment for confounding variables.

### Advantages and limitations

Two main advantages of the present review are the high quality of the reviewed studies and adjustment of the confounding variables such as age, body mass index, and physical activity of the participants. The initial results of the RCTs demonstrated that an increased in daily consumption of vitamin D reduced CVDs mortality [70], and increased vitamin D intake prevented stroke and CVDs [68, 71, 72]. However, it should be noted that there is the possibility of residual confounding in observational studies. Most of the previous and ongoing RCTs have shown that vitamin D supplementation has no impact on CVD surrogate markers in reasonably healthy people [22]. Further evidences by Mendelian randomization trials also did not show any causality of genetically reduced 25(OH)D concentrations and myocardial infarction, ischemic heart disease, or coronary artery disease [73, 74].

This study also has potential limitations like the fact that only prospective cohort studies conducted in less than the last 10 years were reviewed. In addition, the dose-response effect of vitamin D on the incidence and mortality rate of CVDs was not evaluated. This meta-analysis compared the highest and lowest levels of vitamin D and revealed that the highest concentration of vitamin D was associated with increased CVD mortality rates. The heterogeneity in our meta-analysis is partly due to the application of various methods to measure the serum levels of vitamin D. As such, heterogeneity was more significant in studies that used mass spectrometry compared with studies that used radioimmunoassay to measure the serum level of 25-OH-vitD. So that, radioimmunoassay was used as a measure and the other methods were compared to it. Thus, measurement methods not only account for the heterogeneity in the studies but also indicate the important role of measurement tools in epidemiological research.

The findings showed that gender was not a significant factor, which is not supported by previous studies. As such, some studies revealed that vitamin D deficiency was associated with higher CVDs mortality rates in men, whereas in some studies, vitamin D deficiency was associated with higher CVDs mortality rates among female patients. The NHANES III showed that the CVDs mortality was higher among male patients (IRR = 2.38, 95%CI: 1.92–2.96) [75], whereas the Gind study showed the protective role of vitamin D deficiency in female patients (HR = 0.70, 95% CI: 0.57–0.86) [56]. Additionally, Brondumn revealed a higher risk of stroke in female patients (HR = 1.67, 95%CI: 1.30–2.13) [76], which may be due to the limited number of reviewed studies focusing on gender.

The highest category of serum 25-OH-vitD was considered as the reference - i.e. 50 nmol/l in most studies. A majority of the studies reported the adequate level of serum 25-OH-vitD as > 50 nmol/l whereas some studies reported this level as > 70 nmol/l [77–79]. In addition, Bischoff-Ferrari reported the adequate level of serum 25-OH-vitD as 90–100 nmol/l throughout the world [80].

The serum levels of vitamin D vary from 25 to 75 nmol/l in different continents; the serum level was reported as 25 nmol/l in Asia and the Middle East [81] and 40 nmol/l in African-Americans [82].

The Workshop Consensus for Vitamin D Nutritional Guidelines 3 estimated that about 50 and 60% of the older populations in North America and the rest of the world respectively do not have a satisfactory vitamin D status. The consensus further concluded that the situation is similar in younger subjects [83]. Khaw stated that after adjustment for confounding variables, the CVDs mortality rate was lower (11%) in patients with a 25-OH-vitD level of 90 nmol/l than in patients with a 25-OH-vitD level of 30 nmol/l.

Although, there is no clear information on the relationship between serum 25-OH-vitD and life expectancy, experimental studies have shown that vitamin D receptor-knockout mice develop metabolic defects and cardiovascular disorders [84–86]. The present study showed a strong relationship between the follow-up period (longer than five years) and the effect of serum 25-OH-vitD on CVD in cohort studies, which may be due to the varying serum 25-OH-vitD over the long periods, especially in the elderly [68].

Results from a meta-analysis of RCTs also revealed that vitamin D intake was associated with decreased CVDs mortality rate among the elderly; daily vitamin D consumption of 10–20 mg decreased the CVD mortality by 80% [87]. There are still controversies regarding the effect of vitamin D on the reduction of CVD mortality and other illnesses among the meta-analyses of RCT studies [70, 81, 88–91]. Moreover, serum 25-OH-vitD was shown to have a biphasic effect on CVDs, as both increased or decreased serum 25-OH-vitD increased the incidence of CVD [68].

It was not possible to calculate the crude effect because the required data were not available, and the analysis was performed on adjusted effects. Therefore, there is the possibility of residual confounding. However, considering the relatively high-quality data obtained from the studies, the analysis was performed in different subgroups separately.

## Conclusion

This is the first study of its kind that evaluated the relationship between serum 25-OH-vitD status and CVDs using meta-analysis method in the recent years. The findings showed that vitamin D deficiency increased the CVD mortality. Due to the limited number of studies which included patients of both genders, further research is suggested to separately evaluate the effect of vitamin D status on CVDs among men and women.

## Data Availability

The datasets used and/or analysed during the current study are available from the corresponding author on reasonable request**.**

## Abbreviations

CHD: Coronary heart disease
CVDs: Cardiovascular diseases
HR: Hazard ratio
RCT: Randomized controlled trial
REM: Random effect model
RIA: Radioimmunoassay
RR: Risk ratio
